# Prevalence and determinants of evidence of silicosis and impaired lung function among small scale tanzanite miners and the peri-mining community in northern Tanzania

**DOI:** 10.1371/journal.pgph.0002770

**Published:** 2024-09-26

**Authors:** Alexander W. Mbuya, Innocent B. Mboya, Hadija H. Semvua, Simon H. Mamuya, Patrick J. Howlett, Sia E. Msuya

**Affiliations:** 1 Department of Community Health, Institute of Public Health, Kilimanjaro Christian Medical University College, Moshi, Tanzania; 2 Department of Community Health, Kibong’oto Infectious Diseases Hospital, Kilimanjaro, Tanzania; 3 Department of Epidemiology and Biostatistics, Kilimanjaro Christian Medical University College, Moshi, Tanzania; 4 Kilimanjaro Clinical Research Institute, Kilimanjaro Christian Medical Center, Moshi, Tanzania; 5 Department of Environmental and Occupational Health, School of Public Health and Social Sciences, Muhimbili University of Health and Allied Sciences, Dar es Salaam, Tanzania; 6 National Heart & Lung Institute, Imperial College London, London, United Kingdom; 7 Department of Community Health, Kilimanjaro Christian Medical Center, Moshi, Tanzania; Katholieke Universiteit Leuven, BELGIUM

## Abstract

Limited data among miners in Tanzania suggests prevalence of silicosis, obstructive lung disease and restrictive lung disease to be around 1.6%, 1.9% and 8.8% respectively. Our study aimed to determine the prevalence and factors associated with silicosis and impaired lung function among tanzanite mining community in northern Tanzania. We conducted a cross-sectional study, involving 330 miners and 330 peri-mining community members in Mererani mines. Silicosis was defined based on study participants’ history of exposure to mining dust and digital chest radiological findings with reference to the 2011 ILO classification of pneumoconiosis. Impaired lung function was determined by spirometry using American Thoracic Society/European Respiratory Society recommended system 3. Association between evidence of silicosis/impaired lung function and presumed risk factors were determined using binary logistic regression analyses. The study found that 99/330 (30.0%) of miners had silicosis. Total of 65 (9.8%) participants had impaired lung function, of whom 29 (4.4%) had Chronic Obstructive Pulmonary Disease, 32 (4.8%) had restrictive lung disease and 4 (0.6%) had both obstructive and restrictive lung diseases. Unexpectedly, miners who have worked for more than 10years and those worked for 6 to 10 years had 64% (aOR 0.34, CI = 0.17–0.67, p = 0.002) and 48% (aOR 0.52, CI = 0.30–0.89, p = 0.018) lower odds of having silicosis respectively compared those worked for up to 5 years. Participants with more than 10 years of work duration had more than 3-times higher odds of impaired lung function compared to those who had worked for up to 5 years (aOR 3.11, CI = 1.53–6.34, p<0.002). We found a concerningly high prevalence of silicosis despite short durations of exposure to occupational silica dust. Immediate dust control measures including deployment of wet drilling, wearing of personal protective equipment and regular monitoring of dust exposure need to be enforced by the Occupational Safety and Health Authority–Tanzania.

## Introduction

While small scale miners refer to individuals engaging in mining activities of relatively small size in terms of investment and hence mining capacity, artisanal miners refer to people engaging in mining activities that deploy primitive, low technology mechanisms, usually illegal [1]. Silicosis is an ancient, progressive, incurable, but potentially preventable chronic lung disease caused by the inhalation of crystalline silica. The disease occurs following exposure to respirable crystalline mostly in the mining and manufacturing industries. Three forms of silicosis are described although not distinctly defined; acute silicosis, the most histopathologically distinct, may occur following between a few months and up to 5 years of heavy exposure to respirable crystalline silica, accelerated silicosis occurs over 5–10 years of exposure while chronic silicosis occurring over longer periods of exposure, commonly more than 10 years [2–4]. In majority of mining populations, acute silicosis and accelerated disease may be considered rare. Study findings among South Africa (SA) gold miners showed the crude prevalence of chronic silicosis to range between 1.8% to 8.6% [5]. Data on silicosis in Tanzania are scarce but the prevalence among miners is estimated at 1.6% [6]. However, majority of available reports on the burden of silicosis are based on studies conducted among large scale mining populations. Studies of small scale miners have suggested that prevalence of silicosis may be up to 29.1% with shorter periods of heavy exposure to silica dust [7,8]. A recent systematic review found a silicosis prevalence of between 11% and 37% among small-scale miners [9]. Small scale mining activities in Tanzania, including the tanzanite mines in northern Tanzania, are usually unregulated posing significant safety challenges [10]. The Mererani mines in northern Tanzania, are the only mines in the world where valuable tanzanite gemstone is being mined at commercial level, however health and safety practices in these tanzanite mines are well below the international labour standards and workers are constantly exposed to harmful conditions including dust [11,12]. Previous evidence of exposure to high level of mining dust, including respirable crystalline silica (1.23mg/m^3^), which has been associated with occurrence of silicosis and impaired lung function, in the tanzanite mines of Mererani has been documented [11,13] reflecting simple mechanization deploying pneumatic drills, air blowers, and explosives, with almost no any form of dust reduction measures such as wet drilling being deployed or use of PPE [13]. The tanzanite mines are concentrated in about 6km strip of land with other gemstones including ruby, green garnet, tourmaline and rodlite being mined in other villages within Simanjiro district [14] To the best of our knowledge, the current burden of silicosis amongst tanzanite miners is unknown.

Tuberculosis (TB) is common among mining communities, and silicosis has been shown to significantly increase the risk of tuberculosis, particularly in the context of Human Immunodeficiency Virus (HIV) [15,16]. Little is known about tuberculosis rates among small scale miners, however some evidence available from Ghana suggests the prevalence is high [17] and also recent report among small scale miners in Mererani has shown the prevalence to be around 7.0% [18]. Impaired lung function comprises of obstructive lung disease and restrictive lung diseases. Both obstructive lung disease and restrictive lung diseases are associated with occupational dust exposure [19].

This study aimed to determine the prevalence, and factors associated with evidence of silicosis and impaired lung function among tanzanite mining community in northern Tanzania.

## Materials and methods

### Study design and study site

We have previously described in details our study methods [18]. In brief however, we conducted a cross-sectional study in Mererani mines located in Simanjiro district, in northern Tanzania. Simanjiro District is estimated to have a population of 221,211 based on national census of 2012 and a population growth rate of 2.4% [20], with a little more than half being located in Mererani ward where the tanzanite mines are found. Mererani is the only place in the world where tanzanite gemstone is being mined at a commercial scale. Mererani town is located about 5 kilometres from the mines and 70 kilometres from Arusha City. The tanzanite mines in Mererani are all fenced with only one, guarded entrance gate. The weather in Mererani area is generally dry and strong winds with frequent change of directions. For this reason, as well as a control group, we chose to evaluate the peri mining communities for dust related diseases.

### Study population

The study recruited men aged 18 years and above from two groups: small scale miners and peri mining community’s members. Miners were defined as an individual who goes down the mining pits and engages in various activities including drilling, blasting and shovelling of rocks. As for this study, only small scale miners engaging with drilling activities were involved, though there is frequent task shifting in which any miner can perform any of the different activities. The peri mining communities were residents of Mererani town engaging in other non-mining activities who have no history of working in the mines. Most miners work shifts last between 10 to 12 hours, but some may go up to 48 hours. Our experience is that all underground miners are men; hence only men were recruited as study participants. Miners in Mererani utilize simple mechanization using handheld pneumatic drills, wheelbarrows & sacks, and diesel-based pulley systems to accomplish various mining tasks. At the time of data collection, there were 100 active mining pits, each with between 70 to 90 miners, hence a maximum of 9,000 active miners.

### Sample size

Based on 16% estimated (from routine clinical practice) prevalence of silicosis among tanzanite miners in Tanzania, the minimum sample size of 206 participants were selected from the miners group based on 0.05 precision level and critical z value of 1.96. After adjusting by +10% for lung function assessment, +10% for TB investigation and addition +30% for data loss, the total sample came to 325, which was approximated to 330 miners. The same number was taken for the peri mining community to make the overall total sample of 660.

### Sampling and data collection procedures

Following ethical clearance and obtaining permission from other organs, we introduced the study to mines’ managers, who agreed to participate. We randomly sampled 22 pits from a coded list of the 100 mining pits. From each selected pit, a coded list of all mine workers was obtained forming the sampling frame. To attain a sample size of 330, and to avoid disruption of ongoing work, we used random sampling to select 15 small scale miners from each pit. This number of 15 small scale miners per pit ensured a reasonable number of workers remained to continue with work while the selected ones visited the clinic for disease evaluation.

For the peri mining communities, a list of 85 registered streets in Mererani town was obtained from the local government authority. From this, a random sample of 22 streets was selected and, in each street, a reference house was established at the street’s main junction with the Kilimanjaro International Airport (KIA) to Mererani highway. Houses (being it a shop, health facility or school) were selected consecutively, and from each selected house, individual participants were selected conveniently (as per inclusion and exclusion criteria) until 15 participants per street (cluster) were reached, making a total of 330 participants. For purposes of comparison with the miners, only men were selected as study participants.

Participants were scheduled into groups of 20 up to 25 individuals and provided with return transport to the Occupational Health Service Center (OHSC) at Kibong’oto Infectious Diseases Hospital (KIDH), located about 60 kilometres from Mererani town. Procedures performed to the study participants at the OHSC included taking each participant through the interview schedule (S1 Text) adapted from Medical Research Council (MRC) United Kingdom (UK) Respiratory Questionnaire (MRC, 1986), digital chest X-ray examination (DRGEM Corporation, Korea) and spirometry test using Easy on-PC (NDD Medizintechnik AG, Zurich, Switzerland). The study radiologist and a physician both with extensive experience of interpreting digital chest radiograph of pneumoconiosis and who have both been trained on the ILO classification of radiographs of pneumoconiosis, separately read and interpreted the chest X-rays of the study participants based on the 2011 ILO Pneumoconiosis Classification System with a 12-points scale (4 categories and 12 sub-categories). Kappa statistic was used to assess the extent of agreement on the diagnosis of silicosis by the two X-readers. Then, jointly the two compared the results and come up to consensus on the final diagnosis for chest radiological examination for all the participants. In the absence of the ability to histopathologically confirm acute silicosis and in keeping with previous research [8], in those with chest radiological evidence of silicosis, participants with less than 10 years of exposure were classified as having accelerated silicosis and those with at least 10 years of exposure as having chronic silicosis.

The spirometer was calibrated twice per day, using a 3L Volume Calibration Syringe (CRC Medical, DMS Limited, UK). Participants with initial obstructive spirometry results were tested for reversibility using salbutamol (short acting B2 agonist). An increase of at least 12% or at least 200mls in FCV and FEV_1_ following the bronchodilator reversibility test was regarded as a significant response. Participants who failed to perform the initial spirometry test or could not produce reliable results, were re-scheduled for other examinations in one-week intervals until reliable results were obtained, meaning achieving the acceptability and repeatability criteria. Acceptability was done by evaluating each blow curve i.e. flow-volume and volume-time graphs for good quality. This include a sharp start with no hesitation, maximum inspiration and expiration, absence of air flow cessation, absence of cough, inspiration during the trace & leaks and exhalation lasting for at least 6 seconds with less than 50mls being exhaled in the last 2 seconds. Repeatability was assessed by checking (from tests that have achieved the acceptability criteria) the best two values of FEV_1_ and FVC to be within 150mls of each other and if FVC is <1litre, then the best values of FEV_1_ and FVC to be within 100mls of each other [21].

Importantly, unlike in routine clinical practice in which only presumptive (those with symptoms) TB cases are asked to produce sputum, in our study all participants were asked to produce sputum for TB investigation, with a positive GeneXpert MTB/RIF (GeneXpert Dx System, Version 4.8, Cepheid, USA) defining a diagnosis for TB. HIV rapid blood tests (SD BIOLINE HIV-1/2 3.0 Standard Diagnostic Inc.) and fasting blood glucose tests (GlucoPlus^TM^, GlucoPlus Inc. Quebec, Canada) were performed. Participants found to have any of the investigated diseases, were enrolled to care and treatment services as per the Ministry of Health (MoH) guidelines.

### Data management and analysis

Paper case report forms were entered into Excel. Analysis was performed using Statistical Package for Social Sciences (SPSS) software (IBM SPSS Statistics Version 27). The primary outcomes were presence/absence of evidence of silicosis and presence/absence of impaired lung function while the secondary outcomes were the forms of silicosis (whether it is accelerated or chronic based on the duration of occupational exposure to respirable crystalline silica among those with evidence of silicosis) and the grades of impaired lung function. As per this paper, silicosis was diagnosed based on two key factors i.e. history of a participant being exposed to occupational silica dust and having radiological digital chest X-ray findings consistent with silicosis. The peri mining communities was also assumed to be exposed to the mining dust based on being near to the mines, the practice of leaving the mined rocks above ground in open air and given the environmental characteristics of few vegetation and frequent winds. Impaired lung function comprised of three disease conditions: Obstructive lung disease, restrictive lung disease and mixed (obstructive and restrictive) lung disease. Reversible airway obstruction was defined as an increase of at least 12% and at least 200mls in either of FEV_1_ or FVC following bronchodilator reversibility tests among those found to have obstructive lung disease while those with no response/or an increase of less than 12% or less than 200mls in FEV_1_ and FVC on bronchodilator reversibility test, were classified as having COPD.

The ATS/ERS recommended system 3 based on defining airways obstruction using FEV_1_/FVC < Lower Limit of Normal (LLN) and Z scores for FEV_1_ to classify severity (grades 1 to 5) of impaired lung function was used in this study. The LLN Z-score of -1.64 was used for diagnosis of both COPD, restrictive lung disease and Mixed COPD/restrictive lung disease patterns, while for diseases grading, the FEV_1_ Z-scores used were: Z-score ≥ -2 (grade 1), -2.5 ≤ Z-score < -2 (grade 2), -3 ≤ Z-score < -2.5 (grade 3), -4 ≤ Z-score < -3 (grade 4) and Z-score < -4 (grade 5).

The association between evidence of silicosis, our primary outcome, and presumed risk factors was determined using a priori logistic regression analysis. The key explanatory variables for inclusion in both models were duration of work in Mererani, history of previous lung disease, TB, smoking and income level. A similar model was developed for impaired lung function. A statistically significant level of p<0.05 was used while p<0.001 was considered as statistically highly significant.

### Ethical consideration

Ethical clearance was obtained from the Kilimanjaro Christian Medical University College (KCMUCo) No. 2416 and the Medical Research Coordinating Committee of the National Institute for Medical Research (NIMR) No: NIMR/HQ/R.8a/Vol.IX/3308. Permission to conduct the study was sought from, and provided by, the Permanent Secretary–Presidents’ Office, Regional Administration & Local Government (PS-PORALG), and the owners/managers for the specific mining pits. Written informed consent, translated in Kiswahili (local) language was obtained from each study participant.

### Inclusivity in global research

Additional information regarding the ethical, cultural, and scientific considerations specific to inclusivity in global research is included in (S2 Text)

## Results

### Demographic and clinical characteristics

The two populations i.e. miners and the peri mining community deferred in several characteristics. Table 1 shows the socio-demographic and clinical characteristics of the two groups of communities. The miners comprised of relatively younger population with a median age of 35 years, compared to the peri mining community with a median of 39 years. The miners had significantly higher proportion of individuals with lower level of education compared to the peri mining community but also miners had significantly lower duration of working in mines with a median duration of 5 years compared to 8.5 years for the peri mining community. When assessing the income level, the miners had significantly lower median daily income of about USD 2.9 compared to the peri mining community who commonly engage in other non-mining activities like small business and official employment who had median income of USD 7.2 per day. Significantly higher proportion of miners (62.1%) had history of previous lung disease compaired to the peri mining community (44.2%). Unexpectedly, a higher proportion of the peri mining community (7.9%) were found to have TB compared 6.1% of the miners, though the difference was not statistically significant. All (100%) of those with evidence of silicosis were miners.

**Table 1:** Socio-demographic and clinical characteristics of the mining communities in Mererani (N = 660).

Characteristics	Total	Small Scale Miners (n = 330)	Peri-Mining Community (n = 330)	*χ*2	p value
**Age (years)**					
Median (IQR)	38 (31.0–45.0)	35.0 (30.0–44.0)	39.0 (32.0–46.0)		
18–30	158 (23.9)	92 (27.8)	66 (20.0)	9.91	0.019
31–40	254 (38.5)	129 (39.1)	125 (37.9)		
41–50	180 (27.3)	84 (25.5)	96 (29.1)		
>50	68 (10.3)	25 (7.6)	43 (13.0)		
**Marital Status**					
Not in relationship	112 (17.0)	63 (19.1)	49 (14.8)	2.11	0.147
In a relationship	548 (83.0)	267 (80.9)	281 (85.2)		
**Education level**					
≤Primary	468 (70.9)	272 (82.4)	196 (59.4)	42.43	0.000
>Primary	192 (29.1)	58 (17.6)	134 (40.6)		
**Duration of work (years)**					
Median (IQR)	7 (5–11)	5.0 (4.0–7.0)	8.5 (6.0–12.0)		
≤5	238 (36.0)	179 (54.2)	59 (17.9)	121.60	0.000
6–10	254 (38.5)	117 (35.5)	137 (41.5)		
>10	168 (25.5)	34 (10.3)	134 (40.6)		
**Income (USD/day)**					
Median (IQR)	4.3 (2.8–8.6)	2.9 (2.2–7.2)	7.2 (4.3–11.5)		
>4.3^a^	432 (65.5)	157 (47.6)	275 (83.3)	93.30	0.000
≤4.3	228 (34.5)	173 (52.4)	55 (16.7)		
**Smoking duration (years)**					
** Median**		10.0 (4.0–15.8)	11.0 (5.0–17.0)		
Smokers	93 (14.1)	60 (18.2)	33 (10.0)	9.12	0.003
Non-smokers	567 (85.9)	270 (81.8)	297 (90.0)		
**Previous Lung Diseases**					
Yes	351 (53.2)	205 (62.1)	146 (44.2)	21.18	0.000
No	309 (46.8)	125 (37.9)	184 (55.8)		
**Tuberculosis**					
Yes	46 (7.0)	20 (6.1)	26 (7.9)	0.84	0.359
No	614 (93.0)	310 (93.9)	304 (92.1)		
**Lung Function Status**					
Normal	595 (90.2)	290 (87.9)	305 (92.4)	6.69	0.083
COPD	29 (4.4)	16 (4.8)	13 (3.9)		
restrictive lung disease	32 (4.8)	20 (6.1)	12 (3.6)		
Mixed Lung Disease	4 (0.6)	4 (1.2)	0 (0.0)		
**HIV status**					
Positive	8 (1.2)	6 (1.8)	2 (0.6)	2.03	0.155
Negative	652 (98.8)	324 (98.2)	328 (99.4)		
**Evidence of silicosis** ^**b**^					
Yes	99 (15.0)	99 (30.0)	0 (0.0)	116.47	0.000
No	561 (85.0)	231 (70.0)	330 (100.0)		

^a^The overall median income of the study participants was USD 4.3 per day

^b^Evidence of silicosis refers to silicosis diagnosed based only on history of occupational exposure to respirable crystalline silica and chest radiological findings by digital X-ray.

### Evidence of silicosis, ILO classification and lung function status

**Fig 1:**
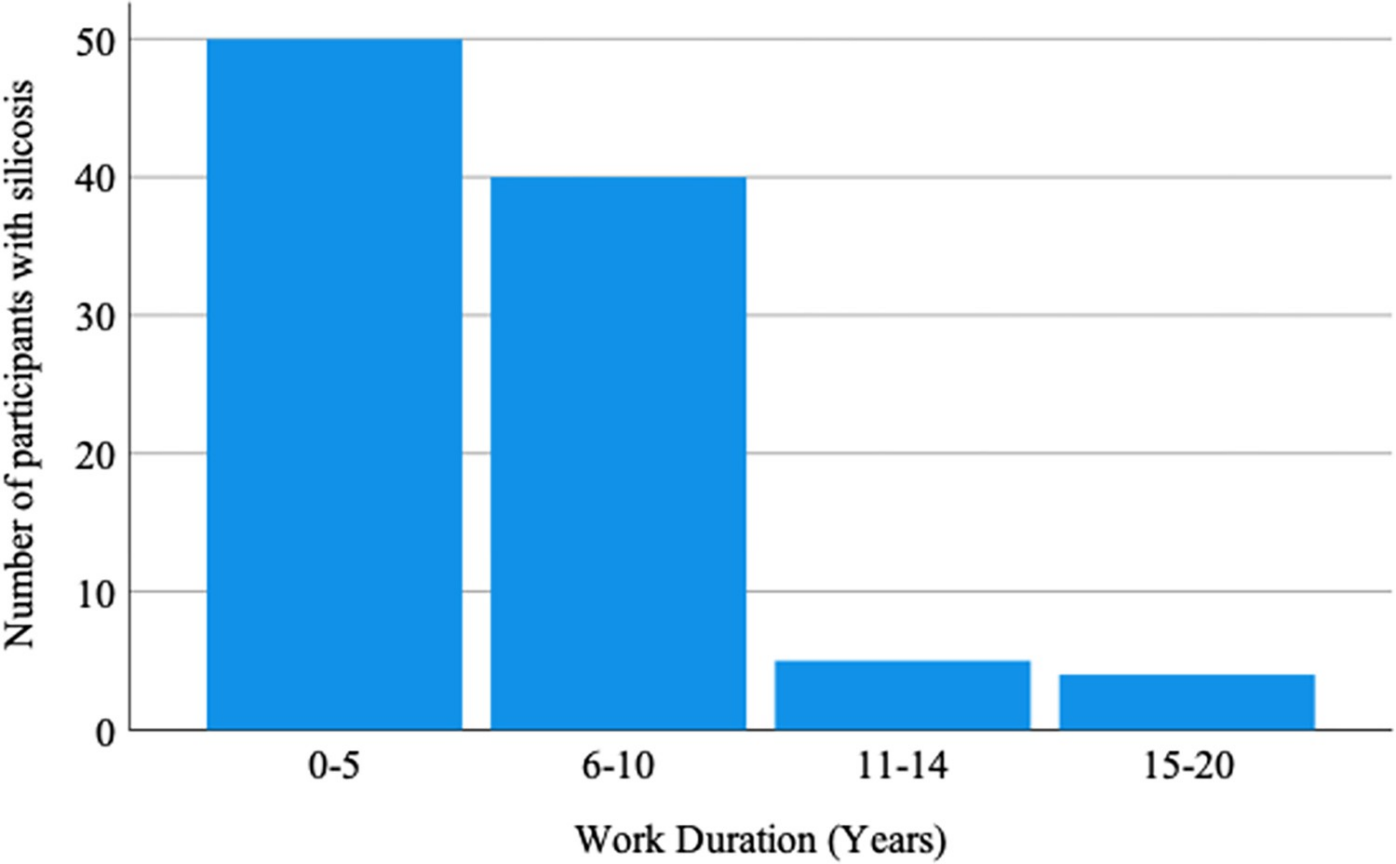
Duration of work in mines among mine workers found to have evidence of silicosis (N = 99). Around 50% had a working duration of up to 5years while around 89% had a working duration of up to 10 years.

X-ray examiner one (radiologist) reported 99 participants (all miners) while the X-ray examiner number two (physician) reported 96 participants (all miners) to have silicosis, with a good agreement between the two readers (Kappa = 0.838). Following a joint review of the chest radiological examination reports by both examiners who read the X-rays, it was finally agreed the number of participants with silicosis to be 99. Among these 99 participants with silicosis, 11 (11.1%) reported to have worked in other mines before shifting to Mererani mines and their respective working duration in other mines were included in the analysis. Fig 1 shows the duration of work in the mines among the miners found to have evidence of silicosis in which among the 99 participants found to have silicosis, 88 (89.9%) reported to have worked in the mines for a duration of up to 10 years, consistent with findings of accelerated silicosis.

Symptoms experienced by the participants included cough 47 (47.5%) in those with silicosis and 97 (42.0%) in those with no silicosis, excessive sweating 23 (23.2%) among those with silicosis and 55 (23.8%) in those with no silicosis, shortness of breath 10 (10.1%) among those with silicosis and 23 (10.0%) among those with no silicosis, weight loos 4 (4.0%) in those with silicosis and 12 (5.2%) in those with no silicosis and bloody stained sputum 1 (1.0%) in those with silicosis and 10 (4.3%) in those with no silicosis.

Of the 99 participants with silicosis, 77 (77.8%) had small opacities while 22 (22.2%) had large opacities. The ILO profusion of 1/0 was taken as the minimum level for the classification. Most small opacities profusions fell under category 2 (2/3) and category 3 (3/2, 3/3 and 3+). Those with profusion below 1/0 were not classified and were combined with those found not to have silicosis under category 0. Majority of those with impaired lung function fell under ILO profusions category 3 (3/3 and 3/+) and large opacities (Table 2).

**Table 2:** The ILO classification of silicosis in relation to the lung function status (N = 660).

Lung function status	Small opacities (Category 1–3)									Large opacities			Category. 0
	**1/0**	**1/1**	**1/2**	**2/1**	**2/2**	**2/3**	**3/2**	**3/3**	**3/+**	**A**	**B**	**C**	
COPD	0	0	0	0	0	1	2	1	2	3	4	2	14
	0.0%	0.0%	0.0%	0.0%	0.0%	7.7%	18.2%	6.3%	20.0%	30.0%	66.7%	33.3%	2.5%
Restrictive lung disease	1	0	2	0	1	2	0	5	2	3	2	0	14
	33.3%	0.0%	40.0%	0.0%	33.3%	15.4%	0.0%	31.3%	20.0%	30.0%	33.3%	0.0%	2.5%
Combined lung disease	0	0	0	0	0	0	0	0	1	1	0	2	0
	0.0%	0.0%	0.0%	0.0%	0.0%	0.0%	0.0%	0.0%	10.0%	10.0%	0.0%	33.3%	0.0%
Normal lung function	2	7	3	9	2	10	9	10	5	3	0	2	533
	66.7%	100.0%	60.0%	100.0%	66.7%	76.9%	81.8%	62.5%	50.0%	30.0%	0.0%	33.3%	95.0%
**Total**	**3**	**7**	**5**	**9**	**3**	**13**	**11**	**16**	**10**	**10**	**6**	**6**	**561**
** **	**100.0%**	**100.0%**	**100.0%**	**100.0%**	**100.0%**	**100.0%**	**100.0%**	**100.0%**	**100.0%**	**100.0%**	**100.0%**	**100.0%**	**100.0%**

**Fig 2:**
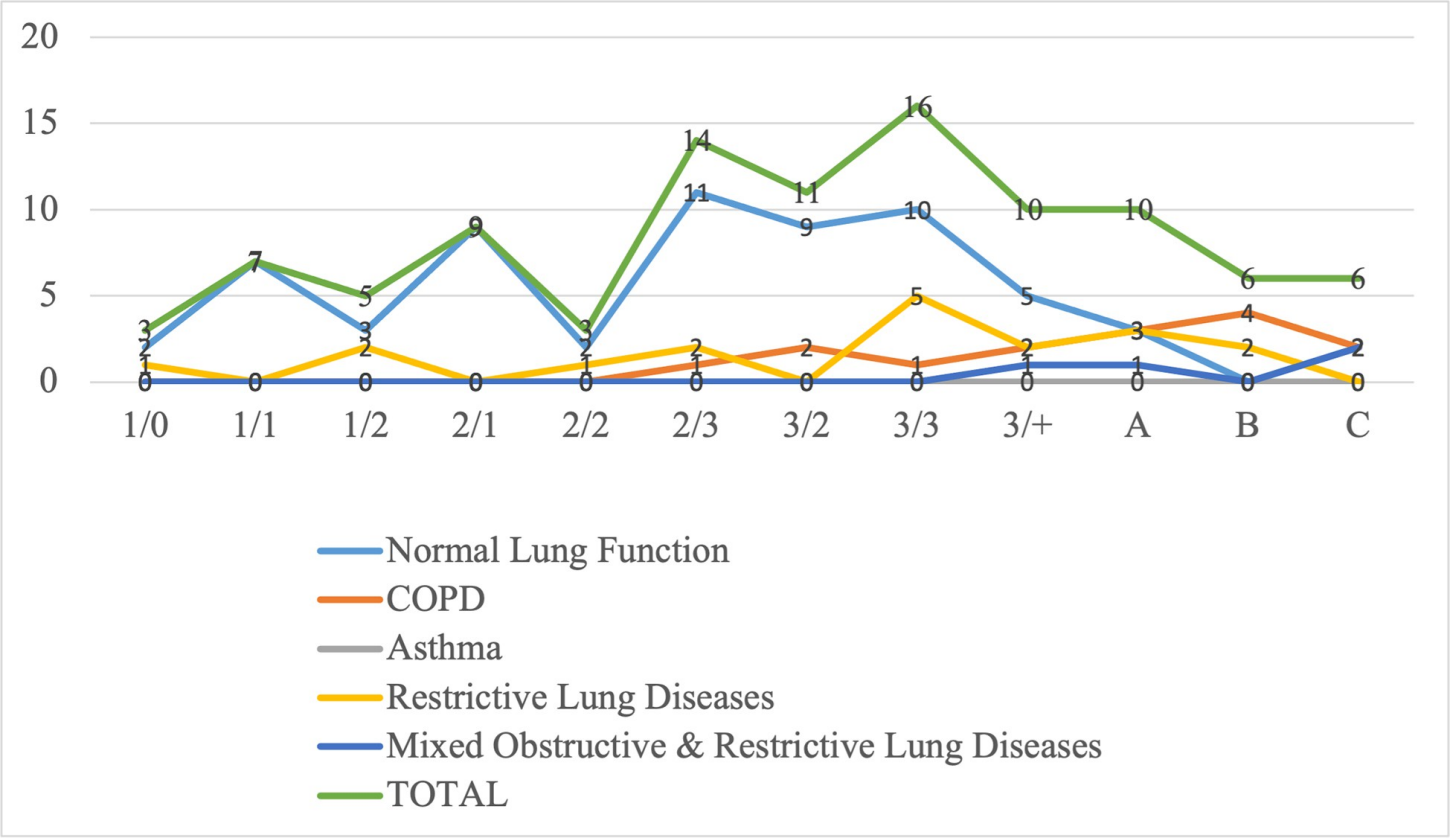
Chart showing the trend of various form of impaired lung function in relation to the ILO classes of silicosis.

Fig 2 shows the trend of ILO classification of silicosis with increasing level of opacities (from) small to large and profusion of small opacities. There is a slight increase in the level of impaired lung functions (restrictive lung disease, obstructive lung disease and combined restrictive and obstructive lung disease) with increasing level of profusions, notably from around ILO profusion 2/1 to large opacity B.

Among the 660 study participants, 65 (9.8%) were found to have various forms of impaired lung function, in which 29 (4.4%) had chronic obstructive lung disease, 32 (4.8%) had restrictive lung disease and 4 (0.6%) had mixed pattern of both obstructive and restrictive lung disease. Of the 65 participants with impaired lung function, 40 (6.1%) were small scale miners and 25 (3.8%) were from the peri mining community. Table 3 shows the lung function status among small scale miners and the peri mining communities based on the LLN Z-score of -1.64 for FVC, FEV_1_ and FEV_1_/FVC. Those found to have obstructive lung disease were subjected to bronchodilator reversibility test however none had a positive test result.

**Table 3:** Lung function status among mining communities in Mererani (660).

Lung Function Status	Total	Miners	Peri-Mining Community
Normal	595 (100.0)	290 (48.2)	305 (51.8)
Restrictive	32 (100.0)	20 (62.5)	12 (37.5)
Mixed	4 (100.0)	4 (100.0)	0 (0.0)
Chronic obstructive	29 (100.0)	16 (55.2)	13 (44.8)

The impaired lung functions were graded based on ATS/ ERS recommended system 3 using Z scores for FEV_1_ in which 46 (70.8%) had grade 1 (mild impaired lung function), 14 (21.5%) had grade 2 (moderate impairment of lung function) and 5 (7.7%) had grade 3 (moderately severe impairment of lung function).

### Factors associated with evidence of silicosis

Crude and adjusted associations between a set of predictors and evidence of silicosis among the mining communities in Mererani are shown in Table 4. Unexpectedly, miners who have worked for more than 10years and those worked for 6 to 10 years had about 64% (aOR 0.34, CI = 0.17–0.67, p = 0.002) and about 48% (aOR 0.52, CI = 0.30–0.89, p = 0.018) lower odds of having silicosis respectively compared to those who have worked for a duration of up to 5 years. Participants with mixed obstructive and restrictive lung diseases had significantly higher odds of silicosis relative to those with no lung function impairment, however the CI was too wide (aOR 17.44, CI = 1.71–178, p = 0.016.

**Table 4:** Factors associated with evidence of silicosis among mining communities in Mererani (N = 660)*.

Variable	cOR (95%CI)	p-value	aOR (95%CI)	p value
**Working duration (years)**				
>10	0.31 (0.16–0.58)	0.000	0.34 (0.17–0.67)	0.002
6–10	0.49 (0.29–0.82)	0.007	0.52 (0.30–0.89)	0.018
≤5	Ref.		Ref.	
**Income (USD/day)**				
>4.3	0.50 (0.33–0.77)	0.002	0.72 (0.45–1.16)	0.177
≤4.3	Ref.		Ref.	
**Smoking status**				
Smokers	0.91 (0.48–1.71)	0.766	0.95 (0.50–1.82)	0.880
Non-smokers	Ref.		Ref.	
**Tuberculosis**				
Yes	0.68 (0.26–1.75)	0.419	0.69 (0.26–1.82)	0.451
No	Ref.		Ref.	
**Lung Function Status**				
Restrictive	1.40 (0.56–3.51)	0.469	1.63 (0.63–4.25)	0.315
Mixed	18.25 (1.88–177.52)	0.012	17.44 (1.71–178.22)	0.016
Obstructive	1.59 (0.63–4.01)	0.329	1.65 (0.63–4.27)	0.306
Normal	Ref.		Ref.	
**HIV status**				
Positive	1.91 (0.38–9.59)	0.433	1.79 (0.34–9.41)	0.489
Negative	Ref.		Ref.	
**Diabetes Mellitus**				
Yes	0.88 (0.33–2.31)	0.795	0.87 (0.32–2.35)	0.780
No	Ref.		Ref.	

*The variable of population i.e. small scale miners and peri mining communities was omitted since none of the participant from the peri mining communities was found to have evidence of silicosis.

### Factors associated with impaired lung function

Table 5 shows the crude and adjusted odds ratios between impaired lung function and a set of predictors. Participants with more than 10 years of work duration had more than 3-times higher odds of impaired lung function compared to those who had worked for up to 5 years, aOR 3.11, CI = 1.53–6.34, p<0.002.

**Table 5:** Factors associated with impaired lung function among mining communities in Mererani (N = 660).

Variable	cOR (95%CI)	p-value	aOR (95%CI)	p value
**Population**				
Miners	1.68 (1.00–2.84)	0.052	1.76 (0.89–3.49)	0.104
Peri-mining community	Ref.		Ref.	
**Working duration i (years)**				
>10	1.73 (0.94–3.19)	0.080	3.11 (1.53–6.34)	0.002
6–10	1.28 (0.68–2.41)	0.440	1.84 (0.95–3.58)	0.072
≤5	Ref.		Ref.	
**Income (USD/day)**				
>4.3	0.62 (0.37–1.05)	0.074	0.56 (0.31–1.02)	0.059
≤4.3	Ref.		Ref.	
**Smoking status**				
Smokers	1.44 (0.74–2.81)	0.288	1.42 (0.71–2.86)	0.324
Non-smokers	Ref.		Ref.	
**Tuberculosis**				
Yes	0.86 (0.30–2.49)	0.786	0.84 (0.29–2.49)	0.756
No	Ref.		Ref.	
**Silicosis**				
Silicosis	11.36 (6.51–19.83)	0.000	1.49 (0.74–3.03)	0.266
No silicosis	Ref.		Ref.	
**Diabetes Mellitus**				
Yes	0.51 (0.12–2.16)	0.359	0.41 (0.09–1.82)	0.241
No	Ref.		Ref.	

The full data set used for the analysis is provided as supporting information (S1 Data).

## Discussion

In light of concerns of high rates of silicosis and impaired lung function, our study aimed to investigate the prevalence of, and risk factors for, silicosis and impaired lung function amongst small scale tanzanite mining community i.e. both miners and peri mining communities. In our study we found high burden of silicosis (30.0%), of whom 89.9% had accelerated silicosis. The prevalence of impaired lung function was 9.8%. Of the 65 participants found to have impaired lung function, 40 (61.5%) were mine workers.

As would be expected from pattern of occupational exposure to respirable dust between these two communities, evidence of silicosis was found only among the small scale miners who are constantly working in congested underground mines and not among the peri mining communities. Though the current study sampled only the drillers but as reported by the miners, there is frequent, tasks shifting, such that any miner can be allocated to undertake any tasks between drilling, blasting, and shovelling. But generally, at any given time around 50% of the miners will be involved with drilling, around 40% involved with shovelling and around 10% work on blasting.

In the current study, significant number of participants reported symptoms related to silicosis though they were not diagnosed with silicosis. This includes cough—97 (42.0%), excessive sweating—55 (23.8%), shortness of breath—23 (10.0%), weight loos—12 (5.2%) and bloody stained sputum—10 (4.3%). With progressive exposure to silica dust, sub-radiological silicosis will progress and be detectable by radiological chest examination. Aside from histopathological evaluation, sub-radiological silicosis may be considered based on evidence of significant exposure to respirable crystalline silica, lack of chest radiological confirmation of silicosis [22] and CC16 levels of below 12ng/ml [23]. Since in the current study CC16 was not investigated, the presence of sub-radiological silicosis could not be confirmed. However, high burden of symptomatic non-silicotic participants highlight the need for monitoring CC16 in this subgroup of miners, though some of them had other diseases with such symptoms, including TB.

Logically it would be expected the burden of silicosis to be higher in miners with longer working duration, however this was not the case with the findings from the current study in which miners who have worked for more than 10years and those who worked for 6 to 10years had 66% and 48% lower odds of silicosis respectively compared to those who have worked for 5 years and below. In the current study, the significantly low proportion of participants with silicosis who have worked for at least 10 years (7.7%) and those who have worked for 6 to 10 years (12.1%) relative to those who worked for up to 5 years (20.9%) could have an effect on such findings. Reports have shown that short periods of intense exposure to respirable crystalline silica to be associated with significantly higher risk of lung diseases, including silicosis [24]. In Mererani tanzanite mines, it is a common practice that once a miner is found to have TB, he will be taken out of the work by the mine’s manager due to fear of transmitting the TB to others but with regard to silicosis, it has been reported by mine workers in Mererani (verbal communication during data collection) that some miners once they are sick to an extent of being unable to work, they will leave the mines to their home residencies and their final fate is generally unknown. This is supported by findings from the current study in which only around 10% of those found to have silicosis, reported to have worked for more than 10 years, implying the likely that most of those who have worked for a longer duration and became sick, have left the mines. A similar pattern of silicosis has been observed among small-scale gold miners in China [8]. Our study showed a silicosis prevalence of 30%, which is towards the higher end though in keeping with other studies of small scale miners [9]. Studies have reported a lower prevalence, including that among stone crushers in Haryana, India of around 6.4% [25] and gold miners in South Africa 19.9% [26]. Our selection criteria meant that current study participants were all engaging in drilling of rocks and given the complete absence of dust reduction mechanisms, including absence of wet drilling and poorly ventilated mining pits, it could explain this high level of silicosis observed. Our finding of high prevalence of silicosis is complemented by the findings of high levels of exposure to respirable crystalline silica among the same current study participants [13]. A study conducted in China among coal workers reported an almost 27% more patients with Coal Workers Pneumoconiosis (CWP) when examined by High Resolution Computed Tomography (HRCT) scan as compared to examination done by Film-Screen Radiography [27]. This difference signifies the variation that may arise because of different diagnostic techniques, hence the possibility of an even higher burden of silicosis finding in the current study if HRCT was used in the diagnosis of silicosis.

Some of the small scale miners from the current study were interviewed about ill health related to the mining activities, in which 85% reported dust to be a major work related health hazard, and 65% of them reported silicosis as a major health challenge [28]. This signifies the need of improving the working environment to ensure mine workers area in a safe condition.

The current study report 9.8% of the participants to have impaired lung function mostly COPD and restrictive lung disease. The high prevalence of impaired lung function among miners could be associated with the occupational exposure to respirable dust. Our group has already demonstrated very high exposures to respirable crystalline silica among miners, but almost none among peri mining communities [13]. This may also be related to a higher prevalence of smoking (18%) among the miners compared to the peri mining communities (11%). The findings of significantly high burden of impaired lung function among the peri mining communities while the exposure to respirable crystalline silica is very low, necessitates further research to address this gap. A pilot study to assess respiratory health among community residing near to gold mines waste in Johannesburg South Africa which defined levels of exposures based on distance from the mine waste as high (home <500m), moderate (500m–1.5km) and low (>1.5km), showed the prevalence of COPD to be more common in the high exposure group (18.6%) relative to the medium exposure (10.2%) and low exposure (10.6%) groups [29]. Compared to the current study, the peri mining communities (located about 5km from the tanzanite mines) will belong to low exposure group residing > 1.5km from the mines’ waste. A study report among non-smokers coal workers (both underground and surface) in USA showed the prevalence of obstructive lung disease of 7.7% which went up to 16.4% among those with CWP [30] while another report among miners in Western Australia reported a prevalence of 6.3% [31].

The current study findings of significant impaired lung function with increased duration of work, especially after 10years, is likely to be related to the cumulative exposure to harmful environment including dust. This finding concur with a study report among iron ore workers in Iran that showed duration of work to significantly lower the FVC, FEV_1_ and FEV_1_/FCV [32]. Opposite findings have been reported from a comparison cross-sectional study among stone crushers in Democratic Republic of Congo (DRC), in which the stone crushers (exposed group) had higher FVC and FEV_1_ compared to the tax drivers, the unexposed group [33] and also a study among underground gold miners in Tanzania that reported history of dust exposure to be not associated with impaired lung function [34].

Mine workers with history of suffering from TB in South Africa were reported to have reduced lung function with FEV_1_ of about 180mls-lower and FVC of about 120mls-lower relative to those who never suffered TB [35]. As reported in our previous study [18], the prevalence of TB among the mining communities in Mererani was around 7.0% which is about 18-times higher compared to the national prevalence of around 0.4% (WHO TB burden estimates 2021; https://www.who.int/teams/global-tuberculosis-programme/data). Unexpectedly, the TB burden was higher (7.9%) in the peri mining communities compared to 6.1% for small scale miners. Given the high burden of TB in both small scale miners and peri mining communities, it is likely that the high prevalence (53.2%) of previous lung disease found in the current study (Table 1), was contributed by previous TB. As has been shown by [36] in their study conducted among adult who have completed TB treatment in Kilimanjaro Tanzania to determine the burden and severity of post tuberculosis lung disease (PTLD), the prevalence of PTLD was found to be 91%.

Limitations in this study included: underpowered study as indicated by the wider confidence interval in Table 4 (lung function status–mixed lung disease). Not utilizing CT scan, which is more sensitive than chest digital X-ray in assessing the pulmonary disease, hence could have missed some of the radiological features associated with silicosis. In addition, for the peri mining communities the specific type of an individual’s work (aside from being non-miner) was not determined, which could have allowed comparison of investigated diseases in different occupational situations. In addition, using a cross-sectional design not allowing followup of assessment.

Silicosis is a major health concern among the small scale miners and impaired lung function is highly prevalent among both small scale miners and the peri mining communities in Mererani, in northern Tanzania. As silicosis is an incurable but potentially preventable condition, immediate dust control measures including deployment of wet drilling, wearing of PPE and regular monitoring of dust exposure should be enforced by OSHA (Tanzania). As per the Occupational Safety and Health Act of 2003 [37], the Chief Inspector should take appropriate corrective measures to mine owners not abiding to the safety and health measures. To facilitate this, Tanzania should join the ILO/WHO global efforts to end silicosis by 2030. Health facilities, especially those serving the mining communities should establish and strengthen expertise in the management of chronic lung diseases, especially COPD. Longitudinal studies focusing to assess TB transmission patterns between miner and the peri mining community should be undertaken.

## Supporting information

S1 Text Interview schedule. (DOCX)

S2 Text Inclusivity in global research checklist. (DOCX)

S1 Data All collected data. (SAV)
