# Supplementary material for: Prevalence and determinants of evidence of silicosis and impaired lung function among small scale tanzanite miners and the peri-mining community in northern Tanzania
Source: PLOS Glob Public Health. 2024 Sep 26;4(9):e0002770. doi: 10.1371/journal.pgph.0002770 (PMC11426446; doi:10.1371/journal.pgph.0002770)
Supplement: S1 Text — (DOCX) [file pgph.0002770.s001.docx]

### Interview Schedule – English Version

**SILICA AND RADON EXPOSURES AND ITS ASSOCIATED EFFECTS ON THE RESPIRATORY SYSTEM AMONG SMALL SCALE MINERS IN MERERANI**

**Interview Schedule – English Version**

**(*Adapted from: Medical Research Council (UK) Respiratory Questionnaire, 1986*)**

**Preamble:** I am going to ask some general questions but also about any breathing or respiratory symptoms.

|  | Interviewer’s ID:  Participant’s ID:  Date of interview: (dd/mm/yyyy) | ____________________  ____________________  ____________________ |  |
| --- | --- | --- | --- |
| **Ser No** | **Question** | **Response** | **Code** |
|  | **LOCATION** |  |  |
| 1 | District |  | L1 |
| 2 | Ward |  | L2 |
| 3 | Village Name |  | L3 |
| 4 | Village ID | \|  \|  \|  \|  \| \| --- \| --- \| --- \| --- \| | L4 |
| 5 | Pit Name |  | L5 |
| 6 | Pit ID | \|  \|  \|  \| \| --- \| --- \| --- \| | L6 |
| 7 | GPS | \|  \|  \|  \|  \|  \|  \|  \|  \| \| --- \| --- \| --- \| --- \| --- \| --- \| --- \| --- \| | L7 |
|  | **DEMOGRAPHY** |  |  |
| 8 | Sex (*record as observed*) | Male: 1  Female: 2 | D1 |
| 9 | How old are you? | Years: _____(*years*) | D2 |
| 10 | What is the highest level of education you completed? | No formal education: 1  Less than primary school: 2  Completed primary school: 3  Completed secondary school: 4  Above secondary school: 5  Refuse to respond: 77 | D3 |
| 11 | What is your region/district of birth? | Region: ___________________  District: ___________________ | D4 |
| 12 | During the past one-year, how many times have you visited your relatives at your region/district of birth? | ________________ | D5 |
| 13 | What is your marital status | Never married: 1  Currently married: 2  Separated: 3  Divorced: 4  Widowed: 5  Cohabiting: 6  Refused to respond: 77 | D6 |
| 14 | For how long have you work as a tanzanite mineworker in Mererani? | ___________________ (years) | D7 |
| 15 | Have you worked as a mineworker in any other areas other than Mererani? | Yes: 1  No: 2 | D8 |
| 16 | ***If yes to Question 15***; for how long have you work as mineworker in other areas other than Mererani? | ___________________ (years) | D9 |
| 17 | What is your average income per month? | ____________________ (Tsh) | D10 |
|  | **SYMPTOMATIC SCREENING OF TUBERCULOSIS** |  |  |
| 18 | Have you had cough for the past two weeks? | Yes: 1  No: 2 | SS1 |
| 19 | Have you had excessive night sweating for the past two weeks? | Yes: 1  No: 2 | SS2 |
| 20 | Have you had significant weight loss (at least 3 kg) in the last 3 months? | Yes: 1  No: 2 | SS3 |
| 21 | Have you noted blood in sputum (or cough up blood)? | Yes: 1  No: 2 | SS4 |
| 22 | Have you had chronic fever for the past two weeks? | Yes: 1  No: 2 | SS5 |
|  | *If the subject is disabled from walking from any condition other than heart and lung disease, please begin questionnaire at* ***Question 27*** |  |  |
|  | **BREATHLESSNESS AND WHEEZING** |  |  |
|  | **During the last month:** |  |  |
| 23 | Are you troubled by shortness of breath when hurrying on level ground or walking up a slight hill? | Yes: 1  No: 2 | B1 |
| 24 | Do you get short of breath walking with other people of your age on level ground? | Yes: 1  No: 2 | B2 |
| 25 | Do you have to stop for breath when walking at your own pace on level ground? | Yes: 1  No: 2 | B3 |
| 26 | If you run, or climb stairs fast do you ever: |  |  |
|  | a) Cough? | Yes: 1  No: 2 | B4a |
|  | b) Wheeze? | Yes: 1  No: 2 | B4b |
|  | c) Get tight in the chest? | Yes: 1  No: 2 | B4c |
| 27 | Is your sleep ever broken: |  |  |
|  | a) By wheeze? | Yes: 1  No: 2 | B5a |
|  | b) Difficulty in breathing? | Yes: 1  No: 2 | B5b |
| 28 | Do you ever wake up in the morning |  |  |
|  | a) With wheeze? | Yes: 1  No: 2 | B6a |
|  | b) With difficulty in breathing? | Yes: 1  No: 2 | B6b |
| 29 | Do you ever wheeze? |  |  |
|  | a) If you are in a smoky room? | Yes: 1  No: 2 | B7a |
|  | b) If you are in a very dusty place? | Yes: 1  No: 2 | B7b |
| 30 | ***If you have answered yes to any of Questions 27-29, are your symptoms better:*** |  |  |
|  | a) At weekends? | Yes: 1  No: 2 | B8a |
|  | b) Extended times when away from work? | Yes: 1  No: 2 | B8b |
| 31 | ***If Yes to Question 30***, please record details of any occupational exposure to other respiratory hazards, e.g., isocyanates, wood dust, or formaldehyde |  | B9 |
|  | **COUGH** |  |  |
| 32 | Do you usually cough first thing in the morning in winter? | Yes: 1  No: 2 | C1 |
| 33 | Do you usually cough during the day – or at night – in the winter? | Yes: 1  No: 2 | C2 |
|  | ***If you have answered yes to Questions 32 or 33***, do you cough like this on most days for as much as three months each year? | Yes: 1  No: 2 | C3 |
|  | **PHLEGM** |  |  |
| 34 | Do you usually bring up phlegm from your chest first thing in the morning in winter? | Yes: 1  No: 2 | P1 |
| 35 | Do you usually bring up any phlegm from your chest during the day – or at night – in winter? | Yes: 1  No: 2 | P2 |
| 36 | ***If you have answered yes to Questions 34 or 35***, do you bring up phlegm like this on most days for as much as three months each year? | Yes: 1  No: 2 | P3 |
|  | **PERIODS OF COUGH AND PHLEGM** |  |  |
| 37 | In the past three years, have you had a period of (increased) cough and phlegm lasting for three weeks or more? | Yes: 1  No: 2 | CP1 |
| 38 | ***If you have answered yes to Question 37***, have you had more than one such episode? | Yes: 1  No: 2 | CP2 |
|  | **CHEST ILLNESSES** |  |  |
| 39 | During the past three years, have you had any chest illness that has kept you from your usual activities for as much as a week? | Yes: 1  No: 2 | CH1 |
| 40 | ***If you have answered yes to Question 39***, Did you bring up more phlegm than usual in any of these illnesses? | Yes: 1  No: 2 | CH2 |
| 41 | ***If you have answered yes to Question 40***, Have you had more than one illness like this in the past three years? | Yes: 1  No: 2 | CH3 |
|  | **PAST ILLNESSES** |  |  |
| 42 | Have you ever had, or been told (at hospital) that you have had: |  |  |
|  | a) An injury, or operation affecting your chest? | Yes: 1  No: 2 | PA1a |
|  | b) Heart trouble? | Yes: 1  No: 2 | PA1b |
|  | c) Bronchitis? | Yes: 1  No: 2 | PA1c |
|  | d) Pneumonia? | Yes: 1  No: 2 | PA1d |
|  | e) Pleurisy? | Yes: 1  No: 2 | PA1e |
|  | f) Asthma? | Yes: 1  No: 2 | PA1f |
|  | g) Other chest trouble | Yes: 1  No: 2 | PA1g |
|  | h) Hay fever | Yes: 1  No: 2 | PA1h |
|  | i) Tuberculosis | Yes: 1  No: 2 | PA1i |
|  | j) Silicosis | Yes: 1  No: 2 | PA1j |
|  | **TOBACCO SMOKING** |  |  |
| 43 | Do you smoke? | Yes: 1  No: 2 | TS1 |
| 44 | ***If you answered No to Question 43***, have you ever smoked as much as one cigarette a day for as long as one year? | Yes: 1  No: 2 | TS2 |
|  | ***If you answered No to Question 43 or 44, omit remaining questions.*** |  |  |
| 45 | How old were you when you started smoking regularly? | ______(*years*) | TS3 |
| 46 | Do (did) you smoke manufactured cigarettes? | Yes: 1  No: 2 | TS4 |
| 47 | ***If you answered yes to Question 46:*** |  |  |
|  | a) How many do you (did) usually smoke per day? | ______ (*actual number*) | TS4a |
|  | b) On weekdays | ______ (*actual number*) | TS4b |
|  | c) At weekends | ______ (*actual number*) | TS4c |
| 48 | Do you smoke any other forms of tobacco? | Yes: 1  No: 2 | TS5 |
| 49 | ***If Yes to Question 48: Record details under additional notes.*** | *Additional notes*:  ____________________________  __________________________  __________________________  __________________________  __________________________  __________________________ | TS6 |
| 50 | When did you give up smoking altogether? | ______________(*month/year*) | TS7 |

***Additional Notes*** *This questionnaire is based on the MRC (UK) Respiratory Questionnaire 1986, which has been extensively validated. This questionnaire will be completed by a worker with the assistance of a native speaker. Additional questions have been added to cover clinical aspects of bronchial hyper responsiveness validated by the Department of Occupational and Environmental Medicine, National Lung Institute. The British Occupational Health Research Foundation (BOHRF) concluded that in the clinical setting questionnaires that identify symptoms of wheeze and/or shortness of breath which improve on days away from work or on holidays have a high sensitivity, but relatively low specificity for occupational asthma.*
